# Size-Dependent Phase Transformation during Gas Atomization Process of Cu–Sn Alloy Powders

**DOI:** 10.3390/ma12020245

**Published:** 2019-01-12

**Authors:** Hao Pan, Hongjun Ji, Meng Liang, Junbo Zhou, Mingyu Li

**Affiliations:** 1 State Key Laboratory of Advanced Welding and Joining, School of Materials Science and Engineering, Harbin Institute of Technology at Shenzhen, Shenzhen 518055, China; panhao@stu.hit.edu.cn (H.P.); lm820gj@163.com (M.L.); jobs_chau@foxmail.com (J.Z.); myli@hit.edu.cn (M.L.); 2 Flexible Printed Electronic Technology Center, Harbin Institute of Technology at Shenzhen, Shenzhen 518055, China

**Keywords:** intermetallics, nucleation and growth, phase transformation, microstructure, high-temperature application

## Abstract

For binary element atomization, it is essential to investigate the phase transformation from liquid to solid as a functions of the droplet sizes, as well as the reaction competitiveness, during gas atomizing solidification of their nuclei. In the present work, a series of phase transformations of undercooled Cu (60.9 wt.%)/Sn droplets were analyzed when atomized by pressure gas. The results indicated that the microstructures of the obtained powders and their morphologies were highly relevant to the droplet size. According to the phase characteristics analyzed by the microstructural observations in combination with the transient nucleation theory, powders with sizes from 10 to 100 μm were divided into three categories, exhibiting lotus-leaf, island, and stripe morphologies. The competitive formation of Cu_6_Sn_5_ or Cu_3_Sn was also controlled by the droplet sizes, and a diameter of approximately 45 μm was identified as the threshold size. After heat treatment at 300 °C for 4 h, the powders consisted of a single η’ Cu_6_Sn_5_ phase. The obtained Cu_6_Sn_5_ phase powders can be used in the field of high-temperature applications as intermetallic balls for integrated chip interconnects.

## 1. Introduction

With the development of powder metallurgy technology, the performance of bulk material was greatly improved by the extensive use of micro and nano powder synthesizing, which stimulates the industrial market demand for powders [1,2,3,4]. The development of powder injection molding in particular further opens the extensive application of powders (e.g., titanium, aluminum, nickel, and steel-based alloy powders) in the fields of aerospace, shipbuilding, automobile, and powder generation industries [5,6]. The metallic parts produced with these powders have advanced properties which differ from those of conventional materials, such as excellent mechanical properties, special magnetic properties, high electrical conductivity and diffusivity, reaction reactivity, and catalytic activity. Studies also reported that highly filled powders can be used in paper-coating processes [7,8]. However, for powders consisting of several elements, their phases and elemental distributions are difficult to control during their formation, which leads to significant impacts on the microstructure and properties of the products produced by additive manufacturing.

Many studies focused on the preparation of micro and nano powders [9,10,11,12,13]. A variety of methods can be applied for powder preparation, but the production process is mainly divided into two categories based on the substantive analysis: mechanical and physical chemistry methods, such as ball milling [9,10], liquid reduction methods [11,12], and atomization [13]. Ball milling takes a long time, and the ball may cause rising temperature due to the impact effect of the grinding, which triggers the metal alloying. Moreover, it is difficult to control the morphology of spherical powders. The reaction of the liquid reduction method affiliates with the chemical reduction reaction and is difficult to control due to the fast reaction rate. In addition, the content of impurities in the powder is too high to purify and the challenge of poor agglomeration of the powders still remains unsolved. It is worth mentioning that gas atomization is a process of pulverizing a liquid metal or alloy stream into small droplets with high-speed airflow before coagulating them into powders. Using the aerosolization method, the droplets are solidified at a higher cooling rate compared to the mechanical method and chemical reduction method, and the atomized powder is spherical and has low oxygen content, which is suitable for the preparation of various kinds of metal and alloy powders.

The prominent advantages of gas atomization already attract much attention. Previously, the aerosol preparation and the application of alloy powders were studied comprehensively. High-efficiency catalyst Al–Ni powders were synthesized via aerosolization [14]. Using gas atomization together with hydrogen annealing, an Ni metal hydride battery AB_5_ alloy could work at low temperature [15]. The electrochemical corrosion behavior of Al–Ni alloy powders was investigated using aerosol spraying [16]. There are also researchers who modeled the process of aerosolization and then measured and controlled the parameters, effectively optimizing the process of aerosolization [17,18]. The microstructure and the phase distribution of the powders were also analyzed extensively. Wang et al. [19] obtained immiscible alloy powders with an egg-type microstructure. Using plasma rotating electrode processing, Guo et al. [20] found that the main phases of Nb–Si-based alloy powders were Nb sosoloid, β-Nb_5_Si_3_, and Nb_3_Si. A fine lamellar eutectic structure was formed in the interior powder during solidification. The phase transformation of the undercooled Ti–48Al (at.%) droplets via gas atomization was also investigated and discussed regarding the primary phases α and β [21].

Though gas atomization is highly regarded, the phase compositions and morphologies as a function of powder size are yet to be addressed systematically. For gas atomization powders with different sizes, the microstructure and the phase composition may be different because of the different solidification conditions. The microstructure evolution of the powders during the gas atomization process also varies. More importantly, an in-depth comprehension of the microstructure evolution during the gas atomization process is critical for controlling the phase composition and surface morphology of the powders, which could promote their potential application in different fields. Therefore, in this paper, we report an approach to the formation of the Cu (60.9 wt.%)/Sn powders using the gas atomization process to control the morphology, microstructural evolution, and phase composition. In particular, the formation mechanism based on powder size is illustrated systematically. Furthermore, the high-temperature applications of atomized powders for chip attachment after heat treatment were also addressed.

## 2. Materials and Methods

**Figure 1:**
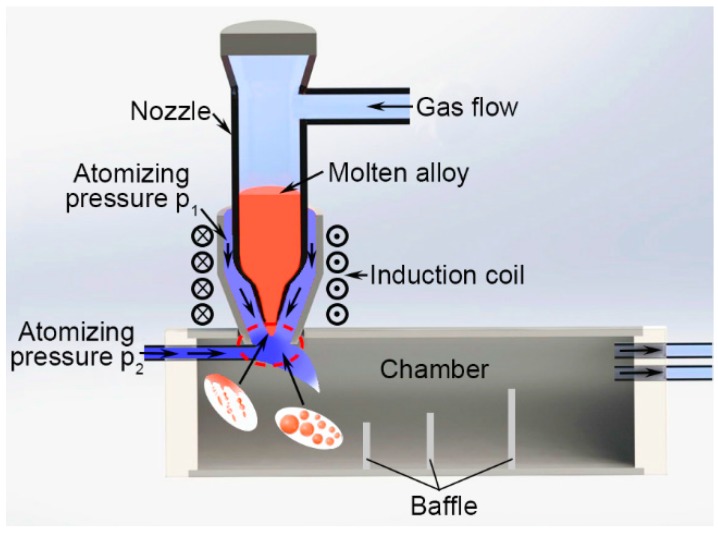
Schematic illustration of the gas atomizer.

A self-designed gas atomizer consisting of a quartz crucible and an atomizing die is shown in Figure 1. The atomizing nozzle is a conical muzzle with an inclined angle of 80° and an outlet diameter of 1.5 mm. Firstly, with a nominal composition of Cu:Sn = 6:5 (at.%), the copper-based alloy was prepared using induction melting of Cu and Sn bars three times in a quartz device with a protective gas in order to achieve a homogeneous composition. Then, the ingot alloy was cut to the appropriate size (1 cm × 1 cm × 2 cm) and heated afterward in the nozzle over its melting point via induction heating after removing the oxide film on the surface of the alloy. The molten alloy was protected by inert gas with slight gas flow at first. After heating for approximately 8 s, the ingot alloy melted, and the gas flow was suddenly increased from 0.15 m^3^/h to 0.5 m^3^/h. The molten alloy stream with the temperature of 600 to 700 °C was removed from the nozzle outlet and atomized using an auxiliary atomization pressure, P_1_, and the dominating atomization pressure, P_2_ (2.3 MPa), through the corundum tube. The kinetic energy transferred from the high velocity of jet-gas to the melt stream caused fragmentation into a variety of shapes down to sub-micrometer sizes, such as flakes, ellipsoids, ligaments, and droplets [22,23]. A series of powders were super-cooled during flying and collected between different baffles. Three different groups of powders ranging from 10 μm to 100 μm in diameter were further classified using standard sieves, whose meshes were 150, 200, 325, and 1800, and then corroded by 10% hydrochloric acid/ethanol solution for 24 h. Finally, powders with different diameters, 74 μm–100 μm, 43 μm–74 μm, and 10 μm–43 μm, were subjected to heat treatment at a temperature of 300 °C for 4 h in an atmosphere of inert gas and then cooled down to the ambient temperature in the furnace to obtain the single Cu_6_Sn_5_ powder.

Phase compositions of the Cu–Sn binary powders with or without thermal treatment were identified by X-ray diffraction (XRD, D/max-2500/PC, Cu Kα, Rigaku, Tokyo, Japan) and differential scanning calorimetry (DSC, STA-449F3, Netzsch, Selb, Germany), together with differential thermal analysis (DTA, STA-449F3, Netzsch, Selb, Germany) testing, under the protection of argon with a heating speed of 10 °C/s and temperature ranging from room temperature to 800 °C. Distribution of different phases as a function of powder size with or without corrosion by 15% hydrochloric acid/ethanol solution was examined using a scanning electron microscope (SEM, S-4700, Hitachi, Tokyo, Japan) equipped with an energy dispersive X-ray spectrometer (EDS, PV7746/40-ME, EDAX, Mahwah, NJ, USA) detector. To observe the interior microstructures of the Cu–Sn binary powders without any treatment, they were mounted in epoxy, polished, and then etched with 10% hydrochloric acid/ethanol solution for 15 s.

## 3. Results and Discussion

### 3.1. Powder Size Distribution and Microstructure Characterization

**Figure 2:**
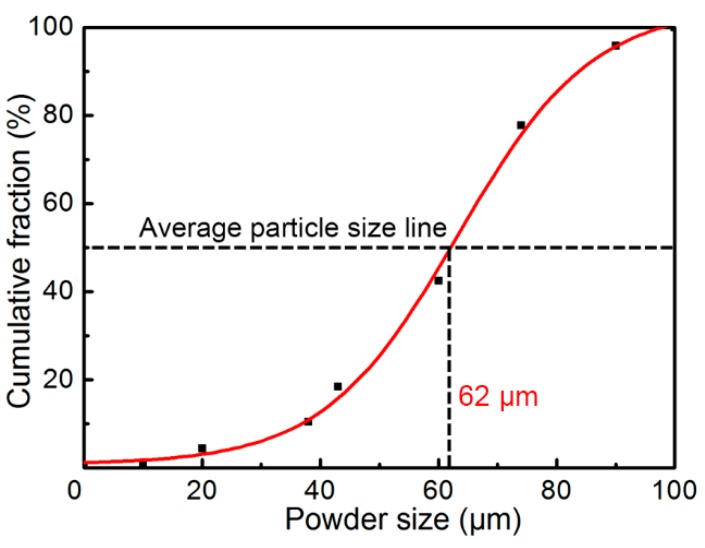
Distribution of powder sizes of gas-atomized Cu (60.9 wt.%)/Sn alloy.

Figure 2 illustrates the cumulative proportion of various sizes of powders. The black dots in the graph represent the true mass fraction measured with the analytical balance after screening with standard sieves whose meshes were 150, 180, 200, 250, 325, 400, 900, and 1800, and the curve was fitted using by the DoseResp function. When the cumulative mass proportion was 50%, the corresponding particle diameter was 62 μm, which is basically the same as the spherical diameter predicted by the equation of Seki et al. [24], since the calculated average particle size was about 65 μm.

*D* = 68*P*^−0.056^ (1)

where *D* is the average powder size (μm) and *P* is the gas pressure (MPa).

**Figure 3:**
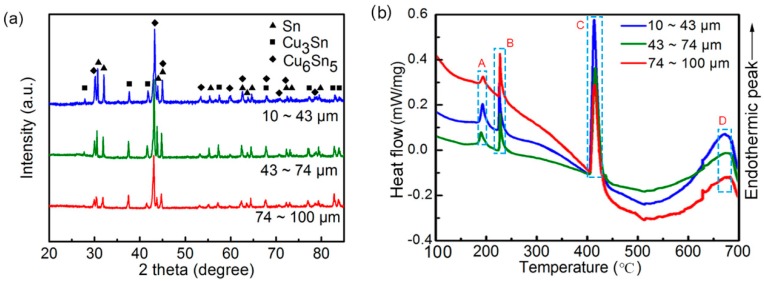
X-ray diffraction (XRD) patterns (**a**), and differential scanning calorimetry (DSC) patterns (**b**) of the atomized Cu–Sn alloy powders with different size.

Figure 3a demonstrates the XRD profile of the obtained powders of different sizes, and the phases mainly consisted of Cu_6_Sn_5_, Cu_3_Sn, and Sn phases, regardless of size. Figure 3b illustrates the DSC profiles of the same weight (0.1 g) of pre-atomized powders with different diameters from 100 °C to 700 °C. When the temperature rose to about 185 °C, an obvious endothermic peak emerged (marked A), indicating the phase transformation from η’ Cu_6_Sn_5_ to η Cu_6_Sn_5_ [25]. The following endothermic peak of melting of Sn (marked B), melting of η Cu_6_Sn_5_ (marked C), and melting of Cu_3_Sn phases (marked D) further proved the existence of the identified phases by XRD. There was an obvious exothermic peak between C and D, indicating bits of the formation of the Cu_3_Sn phase during the heating process of DSC testing. In addition, it is worth noting that, compared to the smaller powders, the bigger ones showed the stronger Sn endothermic peak and weaker Cu_6_Sn_5_ and Cu_3_Sn endothermic peak, indicating that the smaller powders might consist of more Cu_6_Sn_5_ and Cu_3_Sn phases.

**Figure 4:**
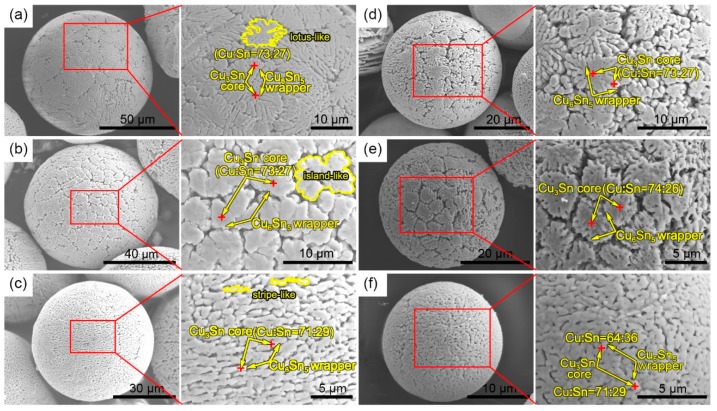
Surface morphology and microstructures of powders with different diameters after being etched with a 10% hydrochloric acid/ethanol solution for 24 h. The powder sizes were (**a**) 84 μm, (**b**) 72 μm, (**c**) 59 μm, (**d**) 44 μm, (**e**) 36 μm, and (**f**) 19 μm. Corresponding enlarged images and compositions are shown to the right.

Figure 4 shows the surface morphology of the microstructure as a function of powder size after deep corrosion for 24 h. The Sn phase on the surface was completely corroded, and only intermetallic phases, such as Cu_6_Sn_5_ and Cu_3_Sn, were residual. In particular, surface morphological features were different between the powders with different diameters. The surface microstructure of the powder with a diameter of 84 μm (Figure 4a) exhibited the shape of a lotus leaf with size ranging from 5 μm to 40 μm. In Figure 4b, the diameter of the powder was 72 μm, whose surface was in an island shape. Some islands were combined to form larger ones. Their sizes ranged from 2 μm to 10 μm in diameter. When the particle size reached approximately 59 μm (Figure 4c), the surface was full of the entirely different stripe-like microstructures, whose sizes were about 0.5 μm in width and 1.5–2.5 μm in length. Especially notable was the fact that, when the particle sizes were down to 44 μm (Figure 4d), 36 μm (Figure 4e), and 19 μm (Figure 4f), the surface microstructures of the powders were in the shapes of a lotus leaf, island, and stripe, respectively, appearing as a reflection of the powders of 84-μm, 72-μm, and 59-μm sizes. Most EDS results of the powder with different diameter showed that the ratio of Cu to Sn was slightly smaller than 3:1 (the EDS results of this paper are the atomic ratio of Cu and Sn, and if the difference between values was less than 4%, the result took the average), indicating that most of the regions marked by a red cross were Cu_3_Sn and a bit of Cu_6_Sn_5_. The Cu_3_Sn core was wrapped with a minority of the Cu_6_Sn_5_, shell because the Cu_3_Sn precipitated before the Cu_6_Sn_5_ during solidification.

**Figure 5:**
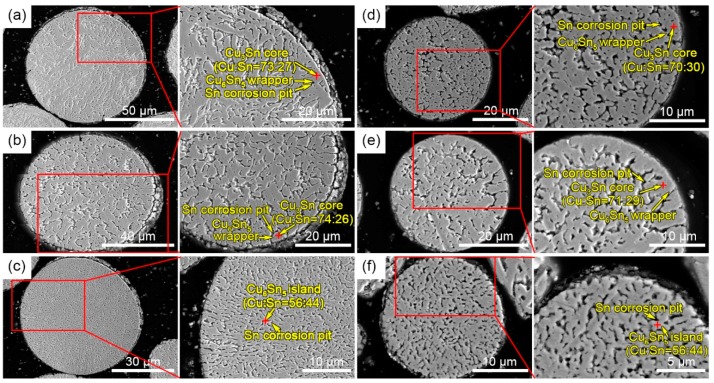
Interior microstructures of powders with different diameters with sizes of (**a**) 82 μm, (**b**) 70 μm, (**c**) 59 μm, (**d**) 38 μm, (**e**) 34 μm, and (**f**) 22 μm. Corresponding enlarged images and compositions are shown to the right.

Figure 5 exhibits the interior morphology of the microstructure of the powders with different diameters after etching with hydrochloric acid/alcohol solution for 15 s. In Figure 5a, the interior of the powder with the diameter of 82 μm exhibited a clear branch-like microstructure and columnar crystals, separated by the Sn phase; the branch-like tissue may correspond to the cross-section of the Cu_6_Sn_5_ wrapper (the outer ring of lotus-like tissue in Figure 4a). Furthermore, the direction of the columnar crystals was generally the same, spanning from the surface to the center. The size of the columnar crystals was about 1.5–2.5 μm in width and 7–10 μm in length, indicating the cross-section of the lotus-like tissue in Figure 4a. In Figure 5b, the microstructure in the powders with the diameter of 70 μm was different from that in Figure 5a and had no feature of directional growth. Meanwhile, the size and the distribution of each island were uniform, similar to the isometric crystal. In Figure 5c, the interior stripe-like microstructure of the powder exhibited the feature of directional growth from the surface to the center and was relatively uniform in size. Similarly, the internal microstructures of the powders with diameters of 38 μm (Figure 5d), 34 μm (Figure 5e), and 22 μm (Figure 5f) were basically the same as those with sizes of 82 μm (Figure 5a), 70 μm (Figure 5b), and 59 μm (Figure 5c), respectively.

**Figure 6:**
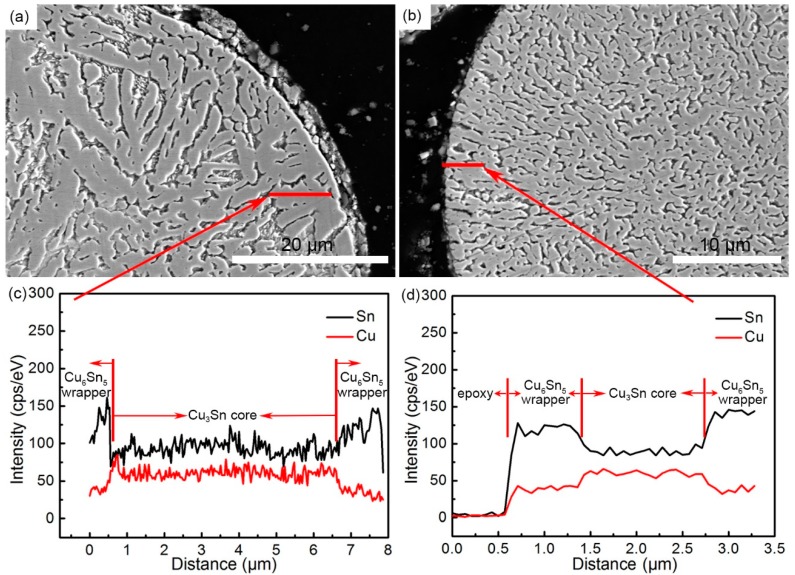
Typical microstructures of cross-sectional powder with sizes of (**a**) 82 μm and (**b**) 59 μm. (**c**,**d**) Corresponding energy-dispersive X-ray spectroscopy (EDS) line-scanning of the marked lines in (**a**,**b**).

Figure 6 illustrates the EDS line-scanning results of the typical cross-sectional microstructures of the powders described in Figure 5a,c. The positions of the lines are shown in Figure 6a,b, and the corresponding line-scanning results are shown in Figure 6c,d. The energy spectrum values of Sn were higher on both sides of the curve, while the middle part was lower. In contrast, the energy spectrum values of Cu were higher in the middle part and lower on both sides (Figure 6c). Meanwhile, the condition in Figure 6d was consistent with that of Figure 6c (the spectrum values of Sn and Cu at the beginning of the curve were almost zero, which proved to be epoxy). Therefore, it indicated that both sides of the line were of the Cu_6_Sn_5_ phase, and the middle part was the Cu_3_Sn phase, which further confirmed the inference that Cu_3_Sn cores were surrounded by the outer Cu_6_Sn_5_ wrapper (Figure 4 and Figure 5).

### 3.2. Formation Mechanism of the Microstructure

**Figure 7:**
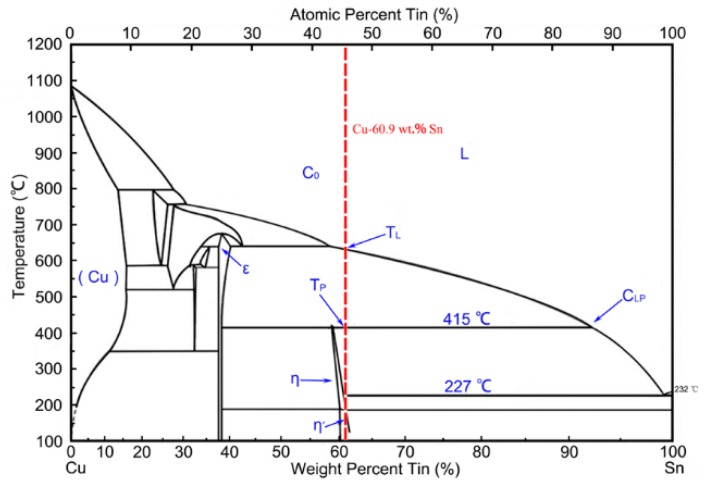
The Cu–Sn phase diagram [27].

Figure 7 illuminates the equilibrium binary Cu–Sn phase diagram. The primary ε phase (Cu_3_Sn phase) firstly precipitated when the liquid melt (L) with a composition of Cu (60.9 wt.%)/Sn (C_0_) was cooled down to the liquidus temperature (T_L_). Then, the initial ε phase grew and continued to precipitate the newly formed ε phase. As the temperature dropped to the peritectic temperature (T_P_), the η phase (η Cu_6_Sn_5_) precipitated on the surface of the ε phase, which was confirmed by the results of unidirectional solidification [26]. Meanwhile, vertical growth along the surface of the Cu_3_Sn phase also occurred because of the peritectic reaction between the ε phase and the liquid Sn [27]. At this moment, the liquid composition was near C_LP_ and there was three-phase (liquid, ε, and η) coexistence in the system.

The proportion of the Cu composition increased with distance from the surface of the ε phase, which would have caused constitutional supercooling ahead of the solidified interface [26]. Therefore, the growth of the ε dendrites required the absorption of the Cu and Sn atoms from the solid/liquid interface. Meanwhile, the growth of the η phase was necessary for it to get access to the undercooled liquid. A secondary η crystal nucleus grew further at the interface of the ε phase, as well as at the three-phase (ε + η + liquid) junctions [27]; however, the newly formed ε phase appeared only between the η crystals because the primary ε phase contacting the liquid would have been consumed. The Cu_6_Sn_5_ wrapper grew at the expense of the primary Cu_3_Sn phase, which demonstrated the occurrence of the peritectic reaction below the peritectic temperature. Though the crystallization was very fast in the atomization process, the analysis above is of great reference value and is consistent with the actual experimental results (Figure 5 and Figure 6).

When the remaining liquid cooled down further, solidification of the eutectic η + β (Sn phase) occurred. The presence of ε + η + β in Figure 4 and Figure 5 (the β phase was corroded) could be explained by the relatively quick cooling of the peritectic solidification, and was concluded by some experiments with different undercooling conditions [28]. Therefore, the heterogeneous nucleation was pivotal in the solidification of the gas-atomized Cu (60.9 wt.%)/Sn powders, and the non-equilibrium peritectic solidification mechanisms played an important role in this process. In addition, multiple nucleation sites were found in Figure 6, which was also reported in gas-atomized Al–Fe–(V, Si) powders [29] and quickly solidified Al–20Si alloy powders [30]. Researchers also reported that the supercooling temperature (ΔT) played a pivotal role in reaction competitiveness and phase selectivity [31,32].

**Figure 8:**
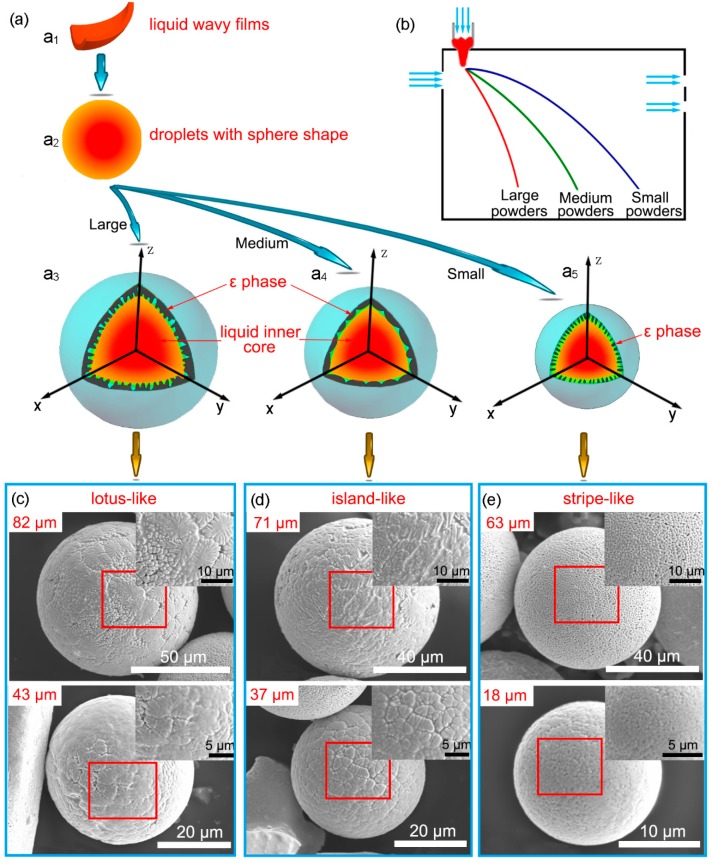
A schematic illustration of the phase transformation mechanism (**a**) as a function of the flying track (**b**); the morphology of powders without corrosion with a series of diameters of (**c**) 82 μm and 43 μm, (**d**) 71 μm and 37 μm, and (**e**) 63 μm and 18 μm.

Combined with the analysis mentioned above, a hypothetical model was constructed to explain the solidification of gas-atomized Cu (60.9 wt.%)/Sn powders. During gas atomization, when the melt was impacted by the cold N_2_, the molten metal was divided into large amounts of unstable melt films, and transformed into wavy films under the gas atmosphere [33]. Figure 8a illustrates the phase transformation model as a function of the flight path (Figure 8b). The wavy films transformed into sphere-shaped droplets (Figure 8a_1_,a_2_) and component segregation in the microdomain of the droplet occurred. Figure 8c–e exhibit the morphology of the powders without corrosion.

The Cu_3_Sn core in the shell of the droplets would have grown inward when the temperature gradient was the largest. When the Cu_3_Sn core grew along the surface and inside to a certain size, the inner liquid phases of the droplets of approximately 84 μm (Figure 4a) were not cooled down to T_P_; thus, the peritectic solidification between the Cu_3_Sn core and the residual liquid phase would not have happened immediately. Instead, the inward growth progressed deeply into the sub-cooled area and continued to grow not only along the original growth direction (the diameter direction of the droplets), but also the direction perpendicular to the original growth direction. However, the supercooling temperature of the liquid between adjacent prominences was lower, such that their crystals protruded more slowly. As the temperature decreased further, the liquid inner core began to crystallize, and the growth of some prominences hindered each other when the outer shell solidified. Faster cooling rates gave rise to shorter crystallization time, resulting in insufficient peritectic reaction, thus yielding the lotus-like microstructure (Figure 4a,d). Therefore, the crystal path model of the droplets of approximately 82 μm and 43 μm was a_1_–a_2_–a_3_–c (Figure 8a,c).

Compared with the droplets of approximately 82 μm and 43 μm (Figure 8c), the growth of the Cu_3_Sn core of the droplets of 37 μm was relatively restricted because its flying distance was farther from the nozzle and the temperature gradient was lower. Thus, the Cu_3_Sn core could grow along the surface and inside the droplet to a certain size until the temperature was reduced to T_P_, followed by incomplete peritectic solidification, as well as solidification of the eutectic η + β. Solidification of their inner cores was slower, such that the inner condensation microstructure was larger in size (Figure 5e,f). The crystallization time was relatively longer, resulting in a relatively adequate peritectic reaction, which would cause the consumption of Cu_3_Sn and Sn. In the same way, the solidification of the droplets of about 71 μm in size was similar to that of the 37-μm droplets (Figure 8d), due to their larger volume though they were closer to the nozzle. Therefore, their crystal path model was a_1_–a_2_–a_4_–d (Figure 8a,d).

For the droplets of approximately 63 μm and 18 μm in size (Figure 8c), the flying distance was the farthest from the nozzle, and the interior peritectic solidification was adequate because its cooling rate was further reduced, which consumed a relatively larger proportion of the ε phase. Then, when the inner temperature dropped below 227 °C, solidification of the eutectic η + β would have occurred after the peritectic solidification. Therefore, their crystal path model was a_1_–a_2_–a_5_–e (Figure 8a,e).

It was worth noting that the flying path of the droplets of about 43 μm in size (Figure 8) was farther away from the nozzle side than that of droplets 63 μm in size (Figure 8). However, the surface of the droplets of about 43 μm in size exhibited a lotus-like tissue, while the other showed a stripe-like tissue. This is because, for the powder of 63 μm in size, the volume factor was more critical in affecting the supercooling temperature (compared with the powder of 43 μm in size), rather than the flying distance. Thus, for the powder of 63 μm in size, a larger volume meant a lower cooling rate, resulting in greater consumption of the ε phase due to the relatively adequate peritectic reaction during solidification. Thus, the stripe-like tissue emerged.

### 3.3. Heat Treatment and Potential Application

**Figure 9:**
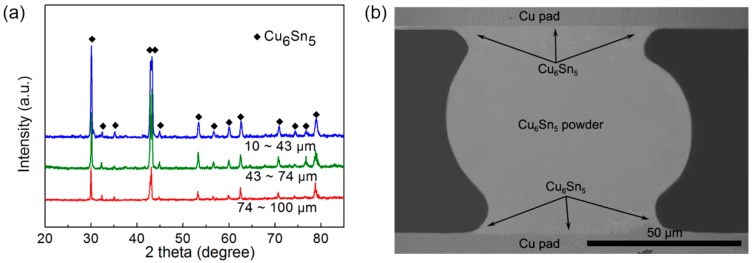
(**a**) XRD patterns of atomized powders as a function of power size after heat treatment at 300 °C for 4 h; (**b**) interconnection application of the Cu_6_Sn_5_ intermetallic powder.

The XRD patterns of atomized powders with different diameters after 300 °C heat treatment for 4 h in an inert atmosphere are shown in Figure 9a. It can be clearly seen that, after long-time heat treatment, the powders consisting of Sn, Cu_3_Sn, and Cu_6_Sn_5_ phases were all transformed into the Cu_6_Sn_5_ phase, which was consistent with the fact that the nominal composition of Cu:Sn in the ingot alloy was 6:5 (at.%). Moreover, the cooling rate of the powders in the furnace was slower than 20 °C/min, such that η–η’ transformation occurred [25], which was also reported in the literature [27].

The obtained pure Cu_6_Sn_5_ powders can be applied in many fields, such as additive manufacturing and three-dimensional (3D) electronic packaging. Here, these Cu_6_Sn_5_ intermetallic powders were applied for high-temperature interconnection to replace the traditional Sn-based solder alloys with low service temperatures. In order to realize the application of the powder, we applied a thin layer of Sn solder paste, which was printed on the two bare copper pads, and then pre-transplanted a Cu_6_Sn_5_ ball with a diameter of 80 μm onto the solder paste under a magnifying glass to form a multi-layer structure of Cu/Sn–Cu_6_Sn_5_–Sn/Cu. The whole structure was then placed in a tube furnace under an inert gas atmosphere at 240 °C for 5 min. The SEM image of the cross-section of the interconnection is shown in Figure 9b. The Sn phase reacted with the Cu pad and was consumed completely in 5 min, leaving the sole intermetallic of Cu_6_Sn_5_ phase in the joint. Thus, such intermetallic interconnections have the potential to be applied in high temperatures (greater than 400 °C). Many researchers found ways to obtain the joints with high melting temperature in a relatively short time. Feng et al. [34] connected Cu substrates with a 50-μm Sn layer (Cu/Sn/Cu sandwich structure) at 260 °C for 4 h, leaving the Cu_6_Sn_5_ and Cu_3_Sn phases in the joint. Yin et al. [35] selected the 10-μm Sn layer as the solder layer, and obtained the full Cu_3_Sn joint at a high temperature of 500 °C for 3 min. Hu et al. [36] used the Sn-coated Cu core–shell particles mixed with flux as the solder layer, and then obtained the joints consisting of Cu_6_Sn_5_ and Cu_3_Sn phases at 250 °C for 12 min. In our experiment, we obtained the Cu_6_Sn_5_ joint at 240 °C for only 5 min and the joint could withstand temperatures over 400 °C. These interconnections, therefore, exhibit considerable potential for application in high-temperature packaging.

## 4. Conclusions

In the present study, the morphology and microstructural evolution of the undercooled Cu–Sn droplets were systematically analyzed. The main conclusions are listed as follows:

(1) The growth of primary phases (Cu_6_Sn_5_ and Cu_3_Sn) were dependent on the droplet size, and the microstructure observation of the primary phase was consistent with the analysis of the transient nucleation theory.

(2) With the decrease in powder size, the microstructures of the powder surface were found to be in lotus-leaf, island, and stripe shapes. The size of 45 μm might be the critical transition diameter. When the size was below 45 μm, the microstructures mentioned above appeared again.

(3) After thermal treatment, the powders were completely composed of a single Cu_6_Sn_5_ phase. Such intermetallic powders can be used in flip-chip packaging. The sole Cu_6_Sn_5_ joint was obtained at 240 °C for only 5 min and could withstand temperatures over 400 °C, exhibiting potential for high-temperature packaging.
